# MetaReg: a platform for modeling, analysis and visualization of biological systems using large-scale experimental data

**DOI:** 10.1186/gb-2008-9-1-r1

**Published:** 2008-01-02

**Authors:** Igor Ulitsky, Irit Gat-Viks, Ron Shamir

**Affiliations:** 1 School of Computer Science, Tel Aviv University, Tel Aviv 69978, Israel; 2 Computational Molecular Biology Department, Max Planck Institute for Molecular Genetics, Ihnestrasse 73, D-14195 Berlin, Germany

## Abstract

MetaReg http://acgt.cs.tau.ac.il/metareg/application.html is a computational tool that models cellular networks and integrates experimental results with such models. MetaReg represents established knowledge about a biological system, available today mostly in informal form in the literature, as probabilistic network models with underlying combinatorial regulatory logic. MetaReg enables contrasting predictions with measurements, model improvements and studying what-if scenarios. By summarizing prior knowledge and providing visual and computational aids, it helps the expert explore and understand her system better.

## Abstract 2

A new computational tool is presented that allows the integration of high-throughput experimental results with the probabilistic modeling of previously obtained information about cellular systems. The tool (MetaReg) is demonstrated on the leucine biosynthesis system in S.cerevisiae.

## Rationale

Given the recent accumulation of high throughput biological data, the task of integrating and analyzing large-scale datasets is a major challenge. A variety of computational modeling approaches have been developed for the analysis of such datasets, such as clustering [1,2] and topological interaction network models [3,4]. While these approaches give a broad, low resolution picture of cellular processes, many biologists are interested in a specific subsystem, and wish to use the results from experiments in order to refine the current knowledge on the system. This analysis of data in the context of the available knowledge is often performed in an informal manner: The researcher sketches a diagram of a relevant subsystem according to the current knowledge. This diagram summarizes and organizes the available knowledge, and assists the expert in analyzing the predicted state of the system in various possible experiments. The predictions are then compared to experimental measurements, and if a discrepancy is found, additional experiments are performed, and the diagram is iteratively refined.

In the case of complex biological systems and massive amounts of data, manual construction of the model, state predictions, comparison with data and systematic model refinements are impractical, and automatic computational methodologies must be employed [5,6]. To address the need for such an analysis workflow, we developed MetaReg, an integrative tool for analysis of steady-state, high-throughput data in the context of specific biological systems. The theoretical foundations of the MetaReg methodology and algorithms are outlined below in the 'MetaReg's algorithmic layer' section (for a complete description, see [7]). While making some gross simplifying assumptions about the behavior of real biological systems, the model was demonstrated to be highly effective on several systems [7-9]. MetaReg enables easy conversion of the current qualitative knowledge on a particular subsystem into a mathematical model, including logical relations among the biological components. The system is represented by a probabilistic graphical model called a Bayesian network [10], which allows distinguishing between regulatory relations that are known at a high level of certainty and those that are more speculative. Given the model, MetaReg predicts the level of each variable under any given genetic perturbation or environmental stimuli. Moreover, MetaReg allows incorporation of high throughput data, and graphical comparison between model predictions and measurements. The most advanced MetaReg capability is suggesting model refinements by systematically seeking changes that increase the fit between model predictions and experimental measurements.

## The MetaReg application

### MetaReg core functionality

**Figure 1:**
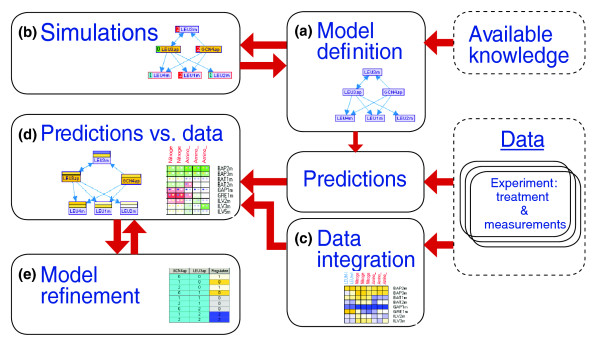
Overview of the workflow in MetaReg. **(a) **The available knowledge about the biological system is represented by a mathematical model. **(b) **The model can be manually improved using simulations. **(c) **The model can be integrated with experimental data. For each experiment we modify the model according to the specific treatment and attach the measurements to the model variables. **(d) **MetaReg predicts the variable states based on the experimental treatment and the predictions are visually compared with the measurements. **(e) **The algorithmic engine proposes refinements to the model in order to increase its consistency with the data. The refinement process can be iterated after accepting certain model changes.

**Figure 2:**
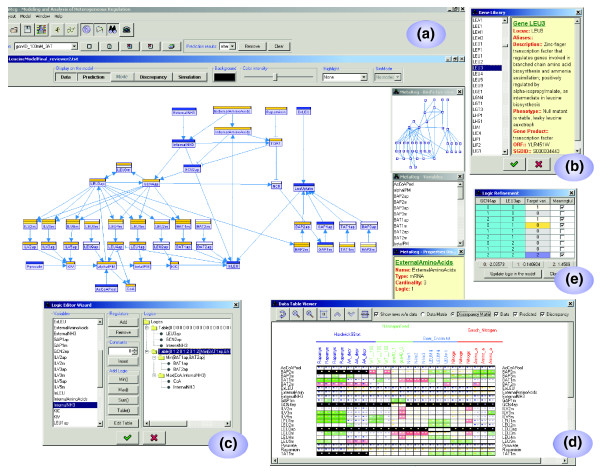
Screenshots of MetaReg core functions. **(a) **The model display. In the main window, the model canvas allows dynamic model layout, model simulation and display of observations (measurements) versus predictions under a particular experiment for each of the model variables. On the right (top to bottom): satellite view of the current model, variable lookup and variable property viewer. **(b) **Selection of variables from a gene database (NCBI Gene or SGD). **(c) **Formulation of a variable's logic using the wizard. **(d) **The discrepancy matrix, which compares predicted and observed levels for all experiments (columns) and variables (rows). **(e) **A logic suggested by MetaReg's model refinement algorithm. The suggestion can be further edited by the user, and incorporated into the model.

Figure 1 illustrates the key features of the MetaReg application and its workflow. The basic workflow begins with model construction and its initial analysis through simulations. Once a current-knowledge model is established, it can be used to predict component values under any experimental treatment (for example, genetic perturbation, growth environment). Next, we compare these predictions to the values observed in the actual experiments under the same treatments, and highlight the discrepancies between them graphically. MetaReg can also automatically refine the model in order to reduce such discrepancies. Screenshots of the main windows from the application are shown in Figure 2. A comprehensive manual of the application is available online [11].

### Model construction

The first step in utilizing MetaReg is the construction of the biological system model on the 'model canvas' (Figure 2a). A MetaReg model consists of a set of biological variables and their regulatory logics. The 'variables' represent different biological entities (mRNA, protein, metabolite, and so on). Each variable may attain several discrete 'states' (three states by default), representing, for example, the transcript level of an mRNA, or the activity level of an enzyme. The state of a variable *v *is influenced by the states of the variables that are connected to *v *by incoming edges. These variables are called the 'regulators' of *v*. Most importantly, every variable is assigned a discrete 'logic', which defines its state given the states of its regulators. For example, if variable A has two activators B and C, its logic might be *Max(B, C)*. We assume all the logics represent steady-state regulatory relations, and thus the model represents the steady-state behavior of the biological system. Every logic is associated with a probability that indicates the certainty in the prior biological knowledge. For example, if a logic is known with high certainty, it will be assigned with a high probability (for example, 90%), and alternative logics will have low probabilities.

The application offers several tools to help in model construction. Variables can be selected from and automatically linked to known databases, such as SGD [12] and NCBI Gene [13] (Figure 2b). Each variable can be attributed with links to relevant journal publications from PubMed, enabling further model curation. The application provides several gadgets for logic definition, including scripting, a tabular editor and a logic wizard (Figure 2c) for hierarchical construction of complex logics. The type of each regulation, activation (→), repression (⊣) or other (-○) is automatically deduced based on the logic of the regulatee (the regulated variable). The model canvas is fully interactive, including capabilities for manual or automatic variable positioning and highlighting of different sets of variables, such as all the metabolites or all the cycles in the model.

### Model simulation

In order to view the behavior of the model in response to different experimental treatments, simulations can be performed. Given a particular experimental treatment, the possible system states are computed as described in [8]. A 'system state' is an assignment of states to all the variables in the model. The user can dynamically design an experimental treatment scenario and visually analyze the system state on the model canvas. If the model contains cycles, several system states might be feasible, and the user can navigate among them.

### Data integration

The application can integrate 'observations' (measurements) from multiple studies. The measured biological components are automatically matched to the model variables. For example, gene expression data are automatically matched to the corresponding mRNA variables, and protein measurements are matched to the corresponding protein variables. As part of the data import, the user must specify the 'experimental treatment' used in each experiment, including the environmental stimulations and genetic perturbations performed in each particular experiment. For example, if the experiment was performed in surplus of nitrogen and on a yeast strain where Leu3 is knocked out, the experimental treatment is 'Leu3 = 0; Nitrogen = 2', where Leu3 and Nitrogen are model variables. Once the data are imported, it is possible to visualize all measured variables under each of the experiments in a single data matrix (Figure 2d; see below), or to view the measurements of a specific experiment projected on the model canvas (Figure 2a).

### Comparing predictions with observations

In order to evaluate the model, the 'predicted' levels of each variable are compared to its 'observed' levels under each experiment. MetaReg provides a prediction engine that infers probabilistically the expected level of each variable in each experiment, given the network model and the experimental treatment (see [7]). MetaReg supports two visualization tools to compare these predictions with the observations, both designed to highlight cases of discrepancies, which are often the starting point of further research. First, the observed and the predicted values for a single experiment can be projected side by side on the model canvas (Figure 2a). The second visualization tool provides a comprehensive view of the discrepancies across all the experiments, in which each cell contains color-coded representation of the observed and the predicted values, along with a representation of the discrepancy between them (Figure 2d). This view allows simple detection of discrepancy 'hot-spots' in which the model fails to explain the data.

### Model refinement

Our methodology enables refinement of the model to obtain better fit between model predictions and observations. The input of the refinement process is the target variable and a set of regulators. MetaReg searches among all possible regulatory logics and outputs the most significant one. The suggested logic can be further edited by the user (Figure 2e). This way the user can test hypotheses about variable regulation.

## Case study: leucine biosynthesis in *Saccharomyces cerevisiae*

### Modeling and simulations

We present a model for leucine biosynthesis and related signaling pathways in *Saccharomyces cerevisiae*. Building on literature reports, we constructed a detailed model of known regulatory relations in this system. The model contains 47 variables (nodes) and 67 regulations (arcs). The model is available from our web site [14].

Leucine is an essential branched-chain amino acid generated from pyruvate via *α*-ketoisovalerate, *α*-isopropylmalate (*α*-IPM) and *β*-IPM in a linear pathway in which nine catalyzing enzymes are involved (Ilv2, Ilv3, Ilv5, Leu9, Leu4, Leu1, Leu2, Bat1, Bat2). The regulation of leucine production is controlled by several known mechanisms [15].

Several leucine biosynthetic enzymes are subject to transcription regulation via the general regulatory pathway of amino acid biosynthesis. Starvation for any amino acid induces the translation of Gcn4 via Gcn2. Gcn4 is a transcriptional activator of enzymes that catalyze several amino acid biosynthesis pathways, including the leucine biosynthetic pathway.

The control of several catalyzing enzymes is regulated by the transcriptional activator Leu3. The activity of Leu3 is regulated by *α*-IPM, an intermediate of the pathway acting as a co-inducer. When *α*-IPM is present, Leu3 acts as activator; when *α*-IPM is absent, Leu3 acts as repressor [15]. Hence, *α*-IPM serves as a sensor of leucine production.

The enzymatic activity of Leu4 is subject to two major controls by metabolites. The first is feedback (end product) inhibition by leucine. At high levels of leucine, Leu4 activity is inhibited, and causes a reduction in the production of the pathway. The second control is inactivation by coenzyme A, a product of the reaction catalyzed by Leu4 and a central energy metabolite in the mitochondria. This control serves as a link between the metabolic process and the energy metabolism context.

**Figure 3:**
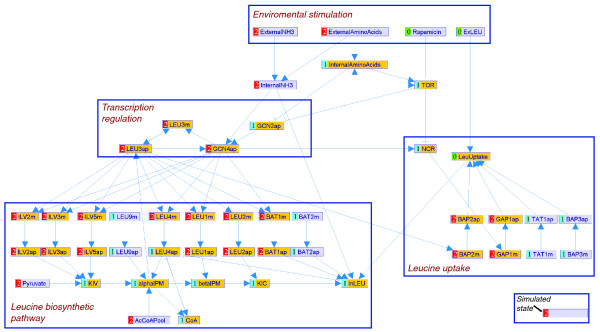
MetaReg model canvas view of leucine biosynthesis in yeast during simulation of leucine starvation. The model includes the extracellular stimuli, leucine uptake into the cell by various permeases, the leucine biosynthetic pathway, and its transcriptional regulation by Leu3 and Gcn4. Variable name suffixes indicate variable types: 'm' represents mRNA and 'ap' represents active protein. Arrows indicate the direction of regulation. Arrow types represent either activation (→), repression (⊣) or other (-○) of a variable; for complex logics, the arrow types are an approximation only. The logics of the regulation are not displayed in this view, but are accessible via other windows (Figure 2c). The model canvas enables highlighting of different sets of variables. In this snapshot, all the cycles in the model are highlighted in orange. The model is presented here during a simulation of leucine starvation: the values of the extracellular stimuli on variables NH_3_, amino acids, rapamycin and leucine were fixed to states 2, 2, 0, and 0, respectively. The resulting predicted (simulated) states of all other variables are presented to the left of their nodes.

In Figure 3, we present a diagram of our model. It includes the leucine biosynthetic pathway, the catalyzing enzymes and their transcriptional control. The state of internal leucine depends on the leucine transport into the cell and on the yield of the leucine biosynthetic pathway. The transport is facilitated via amino acid permeases (Bap2, Bap3, Gap1, Tat1) that are regulated by Gcn4, Leu3, and the TOR signaling pathways. The model includes four environmental stimulators: 'NH_3_^' ^(ammonium), 'rapamycin', 'leucine', and 'amino acids', which indicates availability of all amino acids except leucine that are needed to represent the environmental conditions enforced on the system. The model graph contains many cycles. For example, the general nitrogen control regulation (for example, Gcn4 → biosynthetic enzymes → leucine biosynthesis pathway → internal amino acids → Gcn2 → Gcn4), the leucine-specific transcriptional regulation via Leu3 (Leu3 → biosynthetic enzymes → leucine biosynthesis pathway → *α*-IPM → Leu3), and autoregulation of Leu3 transcription factor (TF) on *LEU3 *gene transcription (LEU3 ↔ Leu3). The variables that are part of cycles in the model are highlighted in Figure 3.

We used three states for each mRNA variable: state '0' represents reduced transcription level compared to the wild type, state '1' represents the wild-type transcription level when cells are grown on YPD medium, and state '2' represents increased transcription level. Similarly, each protein has three states reflecting its activity level (high = '2', medium = '1', low = '0'). The modeling of Leu3 is a special case, since we had to represent its dual role as activator and repressor. We used state '0' for its repressive mode, state '1' represents no effect (for example, in the *leu3 *mutant), and state '2' indicates the Leu3 activator mode. For example, a simulation of the system behavior in leucine starvation is shown in Figure 3.

### Data preparation

We integrated expression profiles from four datasets that contain treatments pertinent to our model: seven profiles in rapamycin treatment after 15, 30, 60 and 120 minutes of incubation and in amino acid deprivation after 1, 1.5 and 2 hours of incubation [16]; six profiles in histidine starvation and various Gcn4 perturbations [17]; six profiles of chemostat growth in nitrogen limiting conditions with and without Leu3 perturbation [18]; and six profiles in nitrogen depletion after 8, 12 and 24 hours of treatment and in amino acid and adenine starvation after 1, 2 and 4 hours of treatment [19]. A complete description of the profiles, the experimental treatments under which they were obtained and the data preprocessing, is available in Additional data file 1.

### Evaluation of the model in accordance with data

**Figure 4:**
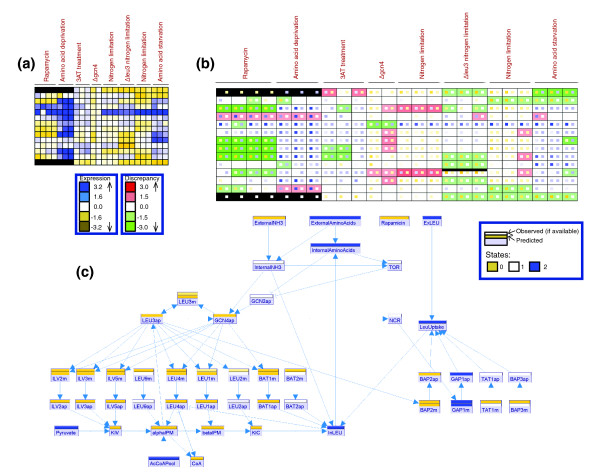
Comparison of measurements and model predictions on the leucine biosynthesis model. The expression levels (both predicted and observed) are indicated in yellow to blue scale (low to high expression). Discrepancies are indicated in green (observed < predicted) to red (observed > predicted) scale. **(a) **The data collected for the leucine biosynthesis model. Rows correspond to mRNA variables and columns correspond to experiments. Cells are colored according to their observed expression levels. Black cells correspond to cases where the mRNA was not measured. The black strip in the top portion of LEU3m cells indicates that it was perturbed in the respective experiments. **(b) **The discrepancy matrix, highlighting differences between measurements and predictions. Rows correspond to mRNA variables and columns to experiments. Each cell contains two squares colored in expression scale, where the left square indicates observed level and the right indicates the predicted level. The background color intensity indicates the discrepancy between the observed and predicted levels. **(c) **The observed and predicted expression levels for a nitrogen limitation experiment eight hours after treatment (Gasch *et al*. [19], matrices A, B column 20) projected on the model canvas as two colored strips above each variable. The strip right above the variable box represents the predicted level. The strip above it (available only for mRNA variables in this case) represents the observed level.

We applied the prediction engine of MetaReg to the collection of experimental treatments described above. The matches and mismatches between the predictions and the observations are displayed by the discrepancy matrix in Figure 4b. While there is a good match for the majority of the components and conditions, the matrix reveals several major discrepancies between the model and the microarray experiments.

The leucine biosynthetic genes *LEU1*, *LEU2*, *LEU4 *and *BAP2 *show an unexpected decrease in expression in the *leu3 *mutant strain (Figure 4b, columns 16-18). The reduction was surprising since Leu3 is known to act as a repressor in these experiments.

In *gcn4 *mutant strains, we observed an increase in the mRNA levels of the leucine biosynthetic genes *BAT1*, *ILV2*, *ILV3 *and *ILV5 *following 3AT treatment (histidine starvation; Figure 4a, columns 11-12). In our model the effect of general amino acid control on these genes is mediated solely by Gcn4. Since Gcn4 is absent in these experiments, our model does not predict such an increase, and a discrepancy appears (Figure 4b, column 12).

For *LEU3*, we observed an increase in expression in two *gcn4 *mutant strains and in nitrogen limitation experiments (Figure 4b, row 11, columns 11-15). According to the literature, *LEU3 *mRNA is upregulated by either Gcn4 or Leu3 TFs. As no amino acid shortage occurs in these experiments, neither Gcn4 nor Leu3 are expected to be active, hence the model predicts a low level of *LEU3 *mRNA, in contradiction to the observed increase.

Following a rapamycin treatment, we observed a consistent decrease in the levels of four biosynthetic genes, *BAT1*, *ILV3*, *ILV5 *and *LEU1*. The effect of rapamycin on the biosynthetic genes is known to be mediated by the TOR pathway through Gcn4 [20]. It is thus expected that under rapamycin treatment, Gcn4 will be active, while Leu3 will not be active. Consequently, the levels of the leucine biosynthesis genes (*LEU1*, *ILV3*, *ILV5*, *BAT1*) regulated by Gcn4 should be alleviated. Surprisingly, we witness a down-regulation of these genes.

For *LEU9*, *BAT2*, *BAP3 *and *TAT1*, we could not find any report on their regulation in the literature, and thus their predicted level is constant. Hence, the discrepancies merely reflect the lack of knowledge about them.

### Leucine model refinement

In order to improve the fit of the model's predictions to the observed data, we used MetaReg's refinement algorithm. We focus here on two representative examples of model refinement. In these examples we suggest improved logics for the way in which Leu3 and Gcn4 jointly regulate *LEU9*, *BAT2 *and *LEU2*.

**Figure 5:**
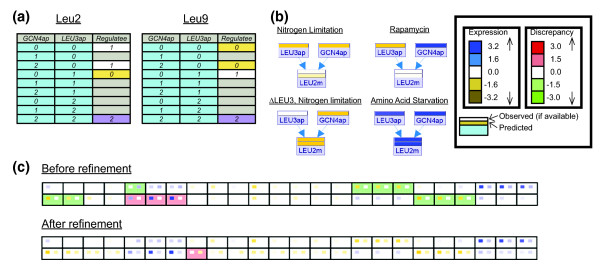
Refinement of the leucine biosynthesis model. **(a) **The refined regulatory logic suggested by MetaReg for LEU2 and LEU9. The regulators of both genes are the transcription factors Gcn4 and Leu3. For each logic, the two columns on the left represent all possible combinations of the regulators' states and the rightmost column is the regulatee's level, colored by an expression scale. Light gray background indicates that the output level predicted for the input combination is not statistically significant. **(b) **Predictions and measurements under specific conditions. MetaReg computes a refined logic based on the regulators' predicted activity level and the observed mRNA level of the regulated gene. As an example, the figure shows the predicted levels of Gcn4 and Leu3 in four conditions along with the measured (top strip) levels of LEU2. The corresponding predicted levels of LEU2 (bottom strip) match the logic suggested by MetaReg for LEU2, as shown in (a). **(c) **Discrepancy matrices for LEU2 and LEU9 before refinement (LEU9 with a constant level; LEU2 activated by Leu3 only) and after refinement (using the logics that appear in (a)). Clearly, the automatic refinement process reduces the disagreement between the model and the measurements.

*LEU9 *and *BAT2 *have similar expression patterns (Figure 4a), but we could not find any report on their regulation in the literature. MetaReg suggests that *LEU9 *is regulated solely by Leu3 with no definite regulatory role for Gcn4 (Figure 5a, LEU9 table, rows 1 and 3). A similar logic is obtained for *BAT2*. Note that for Leu3, MetaReg's refinement matches its known repressive role: when Leu3 acts as a repressor (Leu3 = 0), we observed medium/low transcription of *LEU9*, even though the level of the activator Gcn4 is high (Figure 5a, LEU9 table).

*LEU2 *expression is known to be affected only by Leu3 [15]. Indeed, the suggested logic (Figure 5) shows that the state of Gcn4 does not influence Leu2. As expected, when Leu3 should act as activator (Leu3 = '2') there is high transcription (LEU2 = '2'). However, we do not detect the expected repressive effect of Leu3 on its targets. When Leu3 should act as repressor (Leu3 = '0'), we observe medium *LEU2 *transcription (LEU2 = '1') instead of the expected low transcription.

Figure 5b,c illustrates the refinement process. During refinement, MetaReg tests the predicted activity levels of the TFs (Gcn4 and Leu3) against the observed level of the mRNA in each experiment (Figure 5b), and computes the best logic between the regulators' predicted level and the observations. Consequently, the discrepancies observed for *LEU2 *and *LEU9 *in our initial model (before refinement) are drastically reduced after refinement (Figure 5c).

In the case of *LEU1*, *BAT1*, *LEU4*, *ILV2*, *ILV3 *and *ILV5*, the results were similar to *LEU2 *(not shown). For *BAP2*, *BAP3*, *TAT1 *and *LEU3*, MetaReg did not succeed in deriving a high confidence logical relation, due to inconsistent effects that could not be explained by the model. For example, for *TAT1*, only down-regulation is observed in the data (Figure 4a, last row). For *BAP3*, we observe an inconsistency between two sets of nitrogen depletion experiments in different studies (Figure 4a, columns 13-15 versus 19-21). This probably indicates that each of those genes is regulated by additional elements that are not included in the model.

## MetaReg's algorithmic layer

In this section, we briefly outline the algorithmic layer behind the MetaReg application. A full description can be found in [7].

### Modeling prior knowledge

Our model consists of variables X_1_...X_n_, represented by nodes, and regulations among them, represented by arcs. The set of variables that together regulate variable X_i _are called its 'regulatory unit', denoted Pa_i_. This is the set of nodes that have arcs directed into X_i_. Each variable can be in one of several discrete 'states', and its state in any condition is assumed to be determined by its 'logic', that is, a discrete function of its regulators' states in that condition. Note that this assumption implies that the relevant conditions are in steady state. In order to model our confidence in the prior knowledge, the logic of a variable X_i _is formulated probabilistically as our level of certainty that the variable attains a certain state given the state of its regulatory unit. The uncertainty is modeled by the conditional probability *θ*^i^(X_i _| Pa_i_). This approach allows us to distinguish between regulatory logics that are known at a high level of certainty and those that are more speculative.

The experimental treatment is modeled by fixing the states of each variable that correspond to the environment, and by changing the regulation function priors to reflect the perturbations (for example, when a gene is knocked out, its level is set to zero under that condition, irrespective of the levels of its regulators).

### Data integration

In practice, biological measurements are continuous, and one does not know in advance how to translate them into discrete states. To overcome this, each logical variable X_i _is associated with an observed real-valued variable Y_i_, and the conditional distribution *ψ*^i^(Y_i _| X_i_) specifies the probability of the variable Y_i _to attain a certain observed real value given its state. Hence, *ψ*^i^(Y_i _| X_i_) translates the actual measurements into the discrete model without applying any *a priori *discretization to the data. In MetaReg, each *ψ *is modeled as a mixture of Gaussians.

### The complete computational model

Our probabilistic model defines a 'Bayesian score', which evaluates the fit of the model predictions to the data, measured as the log likelihood of the data given the model:

$$\log\mathrm{Pr}(X,Y|Model)=\log\left(\frac{1}{Z}\prod_{i}\theta^{i}(X_{i}|Pa_{i})\cdot \psi^{i}(Y_{i}|X_{i})\right)$$

where Z is a normalization constant. The conditional probabilities *θ*^i ^are known from our prior knowledge of the biological system, and *ψ *are determined by maximizing a likelihood score using an Expectation-Maximization procedure. This model corresponds to a Bayesian network in the case of acyclic dependencies, or to a factor graph in the more general case where the model contains feedback loops.

### Computing model predictions

The 'predicted level' is the expected value of a variable X_i _given the model and the experimental procedure applied. This is obtained by first computing the posterior states distribution of X_i _using a standard probabilistic inference method called Loopy Belief Propagation [21]. This way we obtain a probabilistic average of all its possible system modes. Then, the (continuous) predicted level of X_i _is its expectation given *θ*^i ^and its states distribution. The comparison of predicted and observed levels (both on the model canvas and in a discrepancy matrix) displays both levels as real values.

### Logic refinement

Given a target gene and its candidate regulatory unit, the refinement process searches in the space of discrete regulatory logics in order to achieve a logic with a locally maximum Bayesian score, while fixing the logics of all other variables. Due to an exponential number of possible logics, we apply a greedy heuristic. In the case of ties the algorithm chooses randomly among the equally scored improvements. The *ψ*^i ^parameters depend strongly on the particular model logics, and thus we re-optimize them using an expectation-maximization (EM)-like procedure during each step of the logical refinement procedure. Note that the refinement process utilizes the Loopy Belief Propagation algorithm, and thus the solution builds on probabilistic averaging of all possible system modes.

## Discussion

MetaReg provides a framework for the modeling and analysis of a biological network *vis-à-vis *high throughput data. A major practical need of molecular biologists today is to generate hypotheses based on network modeling and to iteratively refine the network. MetaReg is designed exactly for this purpose - it allows mathematical modeling of a biological system, interpretation of high throughput data in the context of the prior model, and computational refinement of the model based on the high throughput data. Several other tools with related capabilities, emphasizing visualization or simulations, are being developed (Table 1). The MetaReg platform is unique in its modeling and refinement capabilities, which fit the needs and workflow of biological investigations. It allows streamlined cycles of probabilistic modeling, laboratory experimentation and systematic refinement.

**Table 1:** Available tools related to MetaReg

Tool type	Tools	Description	Relation to MetaReg
Network or model visualization tools	Cytoscape [28] Visant [29] CellDesigner [30] Reviewed in [31]	Tools for constructing visualizations of interaction and regulatory networks. These networks can then be integrated with high-throughput data	These tools offer powerful visualization aids and other analysis aids, but they do not address regulatory logics and do not offer model evaluation or refinement mechanisms
Kinetic and continuous modeling tools	Gepasi [32] BioNetS [33] Dynetica [34] PyBioS [35] Reviewed in [36-38]	Tools allowing detailed dynamical modeling with kinetic parameters and differential equations	These tools can perform detailed model analysis by accurate dynamical simulations, but they cannot discover new mechanisms and rely on detailed mechanistic understanding of the system^1^
Logical modeling tools	BIOCHAM [39] Bionet [40] CellNetAnalyzer [41] GINsim [42]	Tools for modeling regulatory systems using various formalisms, for example, Boolean, discrete, fuzzy logic and so on	Allow model evaluation through simulations, but are not designed for model evaluation and refinement in accordance with high throughput data

^1 ^In several cases the kinetic and continuous modeling tools have parameter optimization capabilities, but only for the given differential equations included in the model. Hence, these tools lack MetaReg's ability to discover unknown mechanism of reaction and changes in model topology. Furthermore, kinetic and continuous modeling approaches require either detailed mechanistic understanding or known model parameters (e.g., reaction constants), which are commonly unknown, even in well studied systems (e.g., the Leucine biosynthesis pathway studied here).

MetaReg is implemented efficiently, computing predictions and logic refinements within a few seconds for 100 nodes, and within an hour for 6,000 nodes (using a network with no more than three regulators per variable, 90% certainty level in all logics, and 100 gene expression profiles). However, the model has practical size limitations: the prediction algorithm run-time increases exponentially with the average number of regulators per variable. Also, for large models with over 300 variables, the automatic layout of the model topology may take several minutes.

MetaReg formalizes the biological system using discrete component states, assuming that the system is in steady state. Clearly these crucial assumptions are a simplification of the biological reality. By making such assumptions, we tried to strike a practical balance between our wish to enable a faithful description of the biological system and the scarcity of accurate knowledge at very high resolution. Indeed, biological processes are inherently temporal, but when the sampling rate (the number and time resolution of experiments) is low relative to the rate of the regulatory mechanisms, we believe that our results here as well as in [7-9] show that the steady state assumption is reasonable.

The accuracy of the prediction and refinement processes may be sensitive to the model size and the certainty in the logics. We have shown previously that the algorithms are highly robust to certainty level on small networks [7]. Indeed, the results shown in the leucine example were obtained using a uniform certainty level of 0.99 for all variables, but we obtained very similar results when using certainty levels of 0.95 and 0.9 (not shown). However, the robustness of our methods to model size and to certainty levels requires further systematic exploration.

A major prerequisite to using MetaReg is formalizing high quality prior knowledge on the pathway of interest. Several efforts to generate databases of curated knowledge on signaling pathway are currently under way (for example, BioModels [22], Reactome [23] and SPIKE [24]). Thanks to such efforts, it will soon be relatively easy to apply the MetaReg methodology in studying many additional biological systems.

### Availability and requirements

Project name: MetaReg (home page at [25]).

Operating system(s): Windows.

Programming language: Java for the envelope and C++ for the algorithms.

Other requirements: Java 1.5 or higher.

License: free for non-commercial users.

Any restrictions to use by non-academics: License needed.

## Abbreviations

EM, expectation maximization; IPM, isopropylmalate; TF, transcription factor.

## Authors' contributions

IU developed the tool, performed the analysis and co-wrote the paper. IG-V conceived the study, developed MetaReg, performed the analysis and co-wrote the paper. RS conceived and supervised the study and co-wrote the paper.

## Additional data files

The following additional data are available with the online version of this paper. Additional data file 1 provides a complete description of the profiles, the experimental treatments under which they were obtained and the data preprocessing.

## Supplementary Material

Additional data file 1 Complete description of the profiles, the experimental treatments under which they were obtained and the data preprocessing. Click here for file
