# Supplementary material for: MetaReg: a platform for modeling, analysis and visualization of biological systems using large-scale experimental data
Source: Genome Biol. 2008 Jan 2;9(1):R1. doi: 10.1186/gb-2008-9-1-r1 (PMC2395235; doi:10.1186/gb-2008-9-1-r1)
Supplement: Additional data file 1 — Complete description of the profiles, the experimental treatments under which they were obtained and the data preprocessing. [file gb-2008-9-1-r1-S1.doc]

**Supplement A - Preprocessing of experimental data**

In experiments [14-17] we used the reported log-ratios between the values under the experimental treatment and under rich medium growth conditions. In experiment [16], where expression under rich medium was not measured, we used the log-ratios between the values under the experimental treatment and under the carbon-limitation experiments. Table S1 lists the experiments used in the order that they appear in **Figure 4** in the main text.

| Subset name | Publication | Profiles | Experimental treatment |
| --- | --- | --- | --- |
| Rapamycin | Harwick *et al.* 14] | 15 min  30 min  60 min  120 min | Rapamycin = 2 |
| Amino acid deprivation | Harwick *et al.* [14] | 60 min  90 min  120 min | ExternalAminoAcids = 0  ExLEU = 0 |
| 3AT treatment | Natarajan *et al.* [15] | Set A  Set B  Set C | ExternalAminoAcids = 0 |
| gcn4Δ | Natarajan *et al.* [15] | 100mM 3AT | GCN4ap = 0 |
|  |  | 0mM 3AT | GCN4ap = 0  ExternalAminoAcids = 0 |
| Nitrogen limitation | Boer *et al.* [16] | Repeat 1  Repeat 2  Repeat 3 | ExternalNH3=0 |
| leu3Δ nitrogen limitation | Boer *et al.* [16] | Repeat 1  Repeat 2  Repeat 3 | LEU3ap=0  ExternalNH3=0 |
| Nitrogen limitation | Gasch *et al.* [17] | 8 hours  12 hours  1 day | ExternalNH3=0 |
| Amino acid starvation | Gasch *et al.* [17] | 1 hours  2 hours  4 hours | ExternalAminoAcids = 0  ExLEU = 0 |

**Table S1**: Experimental Data sources
